# Supplementary material for: Ultrastructural Morphology and Descriptive Analysis of Cuticular Sensilla in Adult Tomicus pilifer (Coleoptera: Curculionidae)
Source: Insects. 2025 Aug 26;16(9):890. doi: 10.3390/insects16090890 (PMC12471193; doi:10.3390/insects16090890)
Supplement: Supplementary file 1 [file insects-16-00890-s001.zip › insects-3803238-supplementary.pdf]

Figure S1. Phylogenetic tree of the 28S rDNA gene constructed using the Maximum Likelihood method.

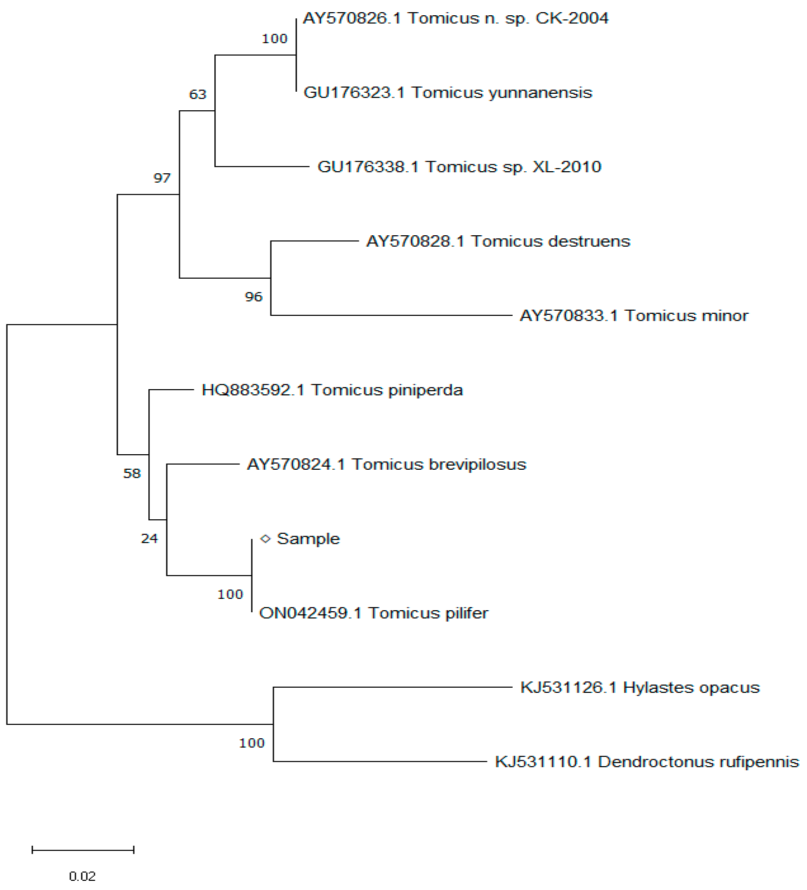

Figure S2. Physiological development of *Tomicus pilifer*.

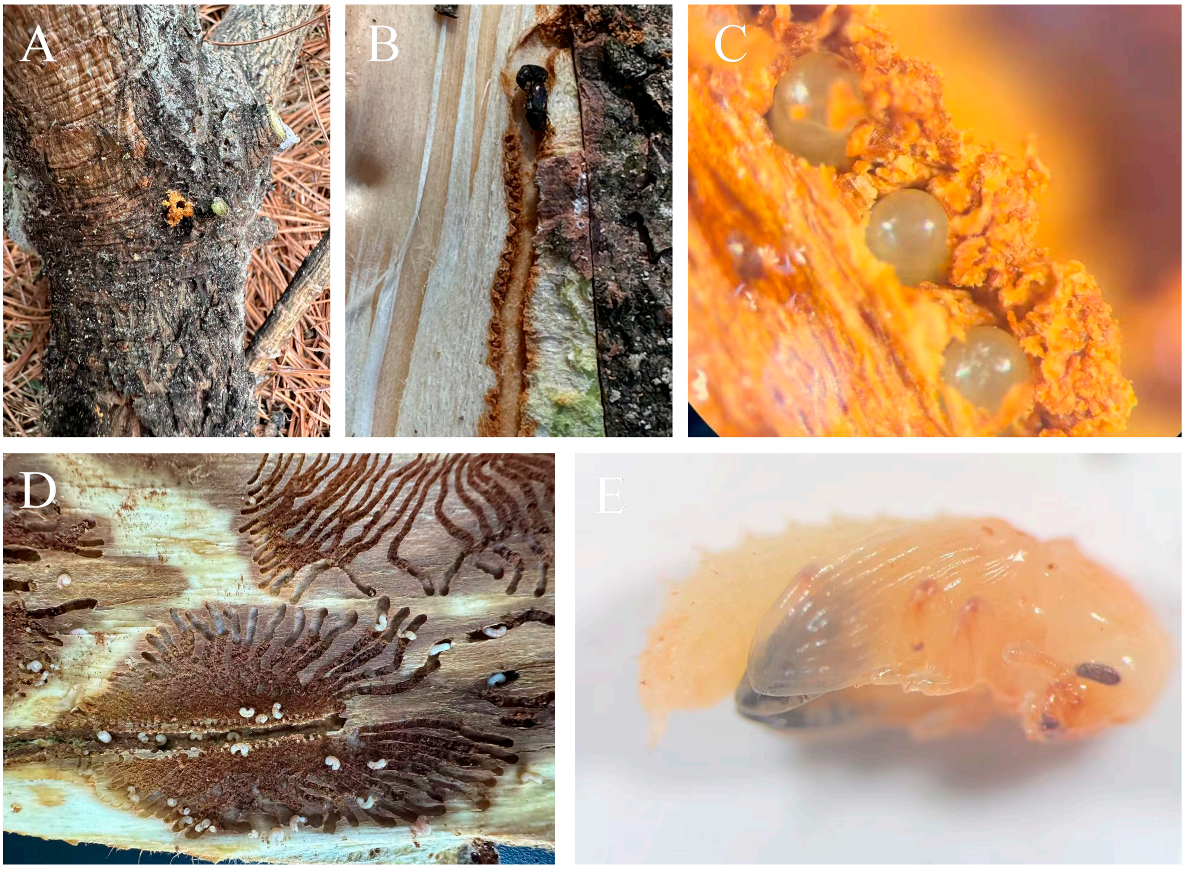

(A) The entrance holes produced by *Tomicus pilifer* on the trunk of *Pinus koraiensis;* **(B)** Adult beetles copulate and oviposit within the galleries; **(C)** The morphology of the eggs and their arrangement within the galleries; **(D)** The intact galleries and the larvae within them; **(E)** The morphology of the pupa.

Table S1. Species and GenBank accession numbers used for DNA sequence analysis in Figure S1.

Species	GenBnak accessions numbers
Tomicus pilifer	ON042459.1
Tomicus brevipilosus	AY570824.1
Tomicus piniperda	HQ883592.1
Tomicus minor	AY570833.1
Tomicus destruens	AY570828.1
Tomicus armandii	GU176338.1
Tomicus yunnanensis	GU176323.1
Tomicus sp. n. CK-2004	AY570826.1
Hylastes opacus	KJ531126.1
Dendroctonus rufipennis	KJ531110.1
